# Multivariate model for cooperation: bridging social physiological compliance and hyperscanning

**DOI:** 10.1093/scan/nsaa119

**Published:** 2020-08-29

**Authors:** Nicolina Sciaraffa, Jieqiong Liu, Pietro Aricò, Gianluca Di Flumeri, Bianca M S Inguscio, Gianluca Borghini, Fabio Babiloni

**Affiliations:** Department of Molecular Medicine, Sapienza University of Rome, Rome, Italy; BrainSigns srl, Rome, Italy; School of Psychology and Cognitive Science, Shanghai Changning-ECNU Mental Health Center, East China Normal University, Shanghai, China; Department of Molecular Medicine, Sapienza University of Rome, Rome, Italy; BrainSigns srl, Rome, Italy; IRCCS Fondazione Santa Lucia, Neuroelectrical Imaging and BCI Lab, Rome, Italy; Department of Molecular Medicine, Sapienza University of Rome, Rome, Italy; BrainSigns srl, Rome, Italy; IRCCS Fondazione Santa Lucia, Neuroelectrical Imaging and BCI Lab, Rome, Italy; BrainSigns srl, Rome, Italy; Department of Sense Organs, Sapienza University of Rome, Rome, Italy; Department of Molecular Medicine, Sapienza University of Rome, Rome, Italy; BrainSigns srl, Rome, Italy; IRCCS Fondazione Santa Lucia, Neuroelectrical Imaging and BCI Lab, Rome, Italy; Department of Molecular Medicine, Sapienza University of Rome, Rome, Italy; BrainSigns srl, Rome, Italy; College of Computer Science and Technology, Hangzhou Dianzi University, Hangzhou Zhejiang Province, People’s Republic of China

**Keywords:** cooperation, social physiological compliance, EEG-hyperscanning, multivariate Granger causality

## Abstract

The neurophysiological analysis of cooperation has evolved over the past 20 years, moving towards the research of common patterns in neurophysiological signals of people interacting. Social physiological compliance (SPC) and hyperscanning represent two frameworks for the joint analysis of autonomic and brain signals, respectively. Each of the two approaches allows to know about a single layer of cooperation according to the nature of these signals: SPC provides information mainly related to emotions, and hyperscanning that related to cognitive aspects. In this work, after the analysis of the state of the art of SPC and hyperscanning, we explored the possibility to unify the two approaches creating a complete neurophysiological model for cooperation considering both affective and cognitive mechanisms We synchronously recorded electrodermal activity, cardiac and brain signals of 14 cooperative dyads. Time series from these signals were extracted, and multivariate Granger causality was computed. The results showed that only when subjects in a dyad cooperate there is a statistically significant causality between the multivariate variables representing each subject. Moreover, the entity of this statistical relationship correlates with the dyad’s performance. Finally, given the novelty of this approach and its exploratory nature, we provided its strengths and limitations.

## Introduction

Cooperation is a complex feature of all social species, including human and non-human animals (Brosnan *et al.*, 2010), which usually points at interaction between actors to reach a common goal (Fehr and Schurtenberger, 2018). This definition partially overlaps with the concept of joint actions (Vesper *et al.*, 2016) which is any kind of social interaction in which subjects share intentions (Fiebich, 2017). This leverages not only on social cognition processes, and in particular on social interaction, but also on cognitive and affective mechanisms of a single subject (Balconi and Vanutelli, 2017b). Recently a three-dimensional model for cooperation based on the cognitive, behavioural and affective axis has been proposed (Fiebich, 2019). According to this model, cooperation is affected by cognitive or emotional alteration in subjects sharing intentions. Moreover, understanding the other’s behaviour is based primarily on social understanding in terms of mental states, known as Theory of Mind (Fiebich, 2019).

The interest in neurophysiological analysis of cooperation was motivated by the will of facilitating team working analysis (Gorman, 2014), overcoming traditional and subjective evaluation methods like interviews and work quality measurements. In fact, cooperation could be positive or negative, according to the results it generates. Difficulties during the team-work generate the difficulty in creating a shared action plan: this could be due, for example, to high demanding situations that impact differently not only on the single subject but on the whole team (Sciaraffa *et al.*, 2017).

What happens if sustaining a subject’s cooperative behaviour becomes too hard because of the task difficulty or of the complexity of understanding other’s behaviour? In this paper, we answer this question investigating the neurophysiological mechanisms underlying cooperation taking into account its multidimensionality.

The past two decades have witnessed the evolution of neurophysiological analysis of cooperation from isolated minds to interactive minds conceptual approaches (Hari and Kujala, 2009; Konvalinka and Roepstorff, 2012; Chatel-Goldman *et al.*, 2013). The main motivation was that cooperative processes intrinsically consist of interaction with the other; therefore, analysis of neurophysiological response during a one-man paradigm could affect the natural response and have some obvious shortcomings (Koike *et al.*, 2015; Redcay and Schilbach, 2019). Whereas the isolated minds theory has a solid theoretical basis and well-defined paradigms, the relatively new approach of interactive minds still has theoretical and practical open issues. In this context, cooperation in the sense of joint activities (Bratman, 1992) is only one of the main fields of investigation, together with the fields of Emotion and Theory of Mind (Chatel-Goldman *et al.*, 2013). This represents a pitfall because examining Emotion, Theory of Mind and Joint actions separately does not allow to be aware of the three-dimensional aspects of cooperation.

### Social physiological compliance

Emotions affect decision-making process, and thus most human choices, including cooperation with others. From a neurophysiological point of view, the effects of emotions could be easily measured because most body parameters such as temperature, heart rate (HR), breathing and sweating are unconsciously controlled by the autonomic nervous system (Kreibig, 2010). Therefore, cardiovascular, electrodermal activity (EDA) and respiratory measurement have been considered as fundamental biomarkers of emotions. In particular, HR and skin conductance level (SCL) are two autonomic variables employed more often than all of the other autonomic variables in literature for the analysis of emotions (Kreibig, 2010). The joint analysis of EDA and HR was born in the framework of social physiological compliance (SPC) (Henning *et al.*, 2001). The physiological compliance was defined in the late 1980s as the psychophysiological change of a joint nature (Smith and Smith, 1987). Until the 1990s, this concept was used to analyse social dynamics: high physiological compliance was associated with high social interaction (Hatfield *et al.*, 1994). The physiological compliance (i.e. the coherence in this case) of EDA, HR, HR variability and breathing of a dyad are predictive of team performance as shown during a tracking task (Henning *et al.*, 2001) and during a building clearing military task of four-person teams (Elkins *et al.*, 2009a). Physiological compliance has been also associated with different shared feelings. Higher synchronization in HR has been correlated with growing trustfulness in dyads (Mitkidis *et al.*, 2015). Higher synchronization in EDA has been found in dyads after receiving positive/negative feedback of their performance as a team during an attention task (Vanutelli *et al.*, 2017) as well as in negative interaction compared to positive interaction in romantic couples (Coutinho *et al.*, 2019). Cooperation has different effects on autonomic measures compared to competition. Previous evidence indicates that EDA synchronization is significantly higher in a cooperative mode, while it is not the same for HR because it has been hypothesized a lower congruence of ECG signals due to different feelings associated with competition (Romero-Martínez *et al.*, 2019). More recently, the efficiency of SPC has been demonstrated using a completely natural task and setting for couples of students (Ahonen *et al.*, 2018). Nevertheless, SPC is not without pitfalls. The measurement of synchronization between physiological data could be due to coincident synchronization; for this reason, it is necessary to use methods to test the statistical significance of synchronization by applying surrogate or bootstrap testing (Coutinho *et al.*, 2019).

### Hyperscanning

Whereas SPC deals with autonomic signals, hyperscanning handles the synchronized acquisition of brain signals of two different subjects (Montague, 2002). The methods, analyses and results of the last 20 years of hyperscanning research have been recently reviewed (Czeszumski *et al.*, 2020). Its first applications included the use of functional magnetic resonance imaging (fMRI). While high spatial resolution of fMRI provides detailed information on deep cerebral areas involved in social interaction, low ecological level and low temporal resolution of this scanning method prevents the acquisition during real interactive tasks. For this reason, the use of EEG technique has been proposed: thanks to its portability it has been possible to analyse two or more people really interacting during ecologic tasks (Poulsen *et al.*, 2017), like, for example, during group interactions in class (Dikker *et al.*, 2017). EEG-hyperscanning applications have shown, essentially through paradigms based on joint actions (Liang *et al.*, 2018), that while two subjects interact, their brain activities are subjected to synchronization (Yi *et al.*, 2018). High inter-brain synchrony has been observed between the right temporoparietal (RTP) and frontal areas during cooperation (Jahng *et al.*, 2017), and in the posterior region of the right middle and superior frontal gyrus during cooperative and obstructive interaction during a Jenga game (Liu *et al.*, 2016). Higher frontal inter-brain synchrony in the theta band has been associated with a mentalizing process in the case of cooperation when members of a couple thought more about their partner’s conduct (Yi *et al.*, 2018), while lower inter-brain synchrony in the beta band has been associated with competitive behaviour during a computerized pong-game (Sinha *et al.*, 2017).

The neural coupling in EEG hyperscanning has been analysed with different methods that could be summarized in three domains: amplitude covariance, phase synchrony and analysis of causality between time series. Several studies have shown that there is covariance in EEG amplitude and power spectra between interacting people (Babiloni *et al.*, 2007; Astolfi *et al.*, 2009). The overlap in activations represents only a weak form of neural coupling, in fact in some cases, similarities of activation could be due to the similarity of common tasks, and certainly, it does not provide information on the temporal dependence that exists between activations of the involved areas (D. Liu *et al.*, 2018). The analysis of phase synchrony and causality could instead provide this information. One of the main used index of phase synchrony in hyperscanning is the Phase Locking Value (Lachaux *et al.*, 1999). This measure of phase consistency is not a measure of information exchange because, as shown by Burgess (2013), phase synchronization may not be due to inherent causes but to coincident synchronization. Unlike this method, causality estimation between time series provides the measure of information exchange in the form of connections between subjects. Multivariate autoregressive model is the most widely used tool to analyse temporal dependencies, to obtain causal relationships, among time series. Time series in the case of EEG-hyperscanning are the brain activities belonging to different subjects collected in a highly synchronized way as if they belonged to the same subject (Babiloni and Astolfi, 2014). However, this approach also has its weaknesses. Whatever the estimator based on multivariate autoregressive models used to assess causality, the accuracy of causality estimation is strongly correlated to the number and size of data segments (Schlogl and Supp, 2006). In addition, EEG signals almost never meet the necessary condition of stationarity and therefore the white noise residual assumption. Non-compliance with the latter is the most common cause of model misspecification and is very common in EEG due to signals cross-talk as an effect of volume conduction (Bressler and Seth, 2011). The effects of volume conduction in the estimation of brain connectivity at the scalp level are represented by spurious connections between time series that cannot be removed. Another important issue is the assessment of statistical significance of the derived connectivity measures. In fact, as for SPC, even for inter-brain causality it is necessary to determine with certainty whether or not exists a connection between two brain areas by means of both empirical and theoretical methods (Baccalá *et al.*, 2013).

Therefore, SPC and hyperscanning approaches showed both pros and cons in cooperation modelling and so far they have been usually employed separately, disregarding the proposed three-dimensional model for cooperation.

This work proposes to fill this gap analysing multivariate Granger causality (GC) of the time series extracted from synchronized recording of EDA, cardiac and EEG signals of subjects during a cooperative task. In fact, based on our hypothesis, cooperation can be seen as the output of a multivariate system caused by interaction between different components (behavioural, affective and cognitive mechanisms) belonging to two subjects.

The behavioural aspect of cooperation in the model is defined by the task. We selected a task that allows subjects to be automatically aware of their performance. The subjects were asked to cooperate in the construction of a Leonardo Da Vinci’s bridge model. Actually, during the task, subjects automatically perceived their performances which, in a closed-loop, affected their cognitive and affective mechanism that affected cooperation and so on. This choice has been made according to the need to introduce a truly interactive and engaging perspective in paradigms of social cognition (Hari and Kujala, 2009).

For the assessment of affective aspects, synchronous time series of cardiac and EDA have been extracted from each dyad. For the assessment of cognitive aspects, EEG data were not used directly, but time series describing brain activity for each subject in a dyad in terms of Neurometrics, i.e. indexes based on the linear combination of spectral power of EEG signals in some frequency bands and certain Regions of Interest (ROIs). In this sense, this work took on an exploratory dimension because different Neurometrics related to cooperative mechanisms have been tested in the model (please refer to the paragraph ROIs selection). Finally, the same approach has been used to analyse cooperation effectiveness.

## Methods

### Participants

Twenty-eight participants (18 female and 2 left-handed, 29 ± 5 years) have been divided into 14 couples (7 couples female-female; 3 couples male-male, 4 couples female-male). All participants were healthy with no history of neurological or psychiatric disorders and with normal or corrected-to-normal vision. Prior to the experiment, each participant was informed about the purpose and contents of the study and provided written informed consent. This experiment was conducted following the principles outlined in the Declaration of Helsinki of 1975 as revised in 2008 and had the permission of the local ethical committee of the Sapienza University of Rome.

### Experimental set-up

**Fig. 1.**
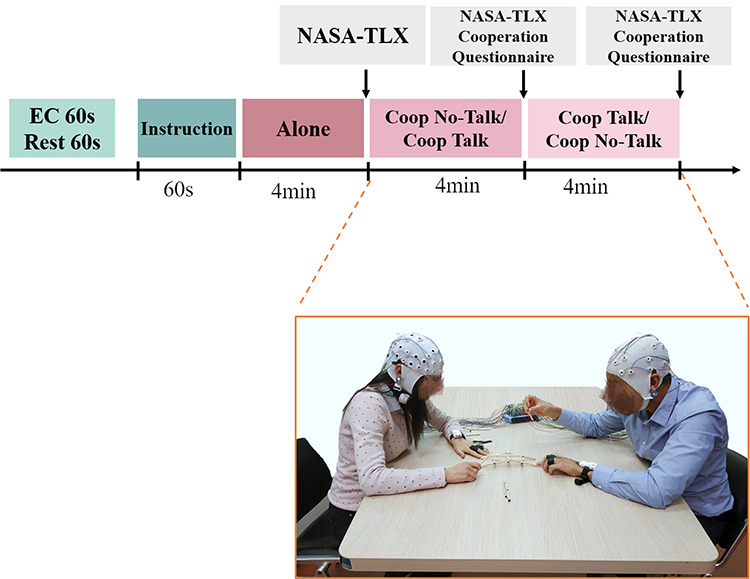
Experimental set-up. Timeline of experimental phases: 60 s of Eye Closed (EC) condition and Rest condition followed by 60 s to read instruction, 4 min to accomplish the Alone condition followed by the 2 cooperative phases. After each condition, questionnaires to evaluate the perceived workload (NASA-TLX) and cooperation have been filled. In the picture, a representation of the cooperative execution of the task where the participants seated in front of each other on opposite sides of a rectangular table while they were constructing a Leonardo’s bridge model.

The experimental protocol consisted in the construction of the Leonardo Da Vinci’s bridge model (Figure 1). This model consists of 15 pieces of three different shapes that must be arranged in order to make the bridge standing. Participants in each dyad were seated in front of each other on opposite sides of a rectangular table. Before the test, participants were asked to seat with their eyes closed for 1 min and then to look at a white sheet for another minute as a baseline value for neurophysiological measurements. After that, the participants were given 1 min to read instructions including a description of every kind of piece to complete Leonardo’s bridge model. The protocol comprised three experimental conditions: Alone, cooperation with forbidden talk (CoopNT) and cooperation with allowed talk (CoopT). The Alone condition was performed firstly for each couple because, according to the results of pilot experiments, this phase is necessary so that subjects can create their own strategy which is a necessary condition for cooperation. The order of the cooperation conditions was counterbalanced across dyads. To mitigate learning effects, different groups of pieces were given to the subjects according to the condition. In the Alone phase, all pieces were given to each participant to complete the bridge model on their own. During the cooperative conditions, the sticks were divided into two parts and given to each participant of a dyad separately. The groups of pieces were not randomly assigned, but balanced division (each participant got the same kind of pieces) has been conducted for the CoopNT condition and unbalanced division (each participant has more or all pieces of one kind) for the CoopT condition. During CoopT condition, the two players can discuss strategy and agree on how to set up the bridge model. During CoopNT condition, the participants could not talk to each other. For each phase, the maximum time available to the subjects was 4 min. This value has been chosen because it was the average completion time during pilot experiments.

### Performance scoring

The performance of each condition has been computed according to the following:

(1) \begin{equation*}Score = 1 - {{{t_{el}}} \over {240}}{{\rm *}}{{w + m} \over {15}}\end{equation*}

that is subtracting from 1 the elapsed time in seconds (${t_{el}}$) divided by the total time available to subjects (240 s) multiplied for the sum of the number of missing ($m$) and wrongly placed pieces ($w$) respect to the total number of pieces (15). The scores of the two cooperative phases have been compared performing a Wilcoxon signed-rank test (α = 0.05).

### Subjective workload and team-workload evaluation

The weighted NASA Task Load Index (NASA-TLX) questionnaire (Hart and Staveland, 1988) has been used to measure the mental workload perceived by subjects, a measure of the amount of cognitive resources required for performing a task. Thanks to this construct we can analyse both how difficult the task was perceived, and the possible presence of learning effects. The NASA-TLX uses six 100-points range subscales to assess mental workload in terms of Mental demand, Physical demand, Temporal demand, Performance, Effort and Frustration. Each of these items is weighted according to a pair comparison procedure (i.e. the number of times each dimension is chosen between two as the most relevant for the workload) that each subject performs before the workload assessments. However, it has been demonstrated that during the cooperative tasks, the workload measurement is affected in a ‘multiplicative or nonlinear’ way by the team itself (Funke *et al.*, 2012). For this reason, the Team Workload Questionnaire has been used for the assessment of team workload (Sellers *et al.*, 2014). This is an extension of NASA-TLX and consists of six 100-points range subscales to assess the team mental workload in terms of Communication demand, Coordination demand, Time Share demand, Team Performance, Team Frustration and Team Support.

Subjective workload has been compared between the three experimental conditions (Alone, CoopNT and CoopT) performing a Friedman Test (α = 0.05). The *post hoc* analysis has been performed by means of Dunn & Sidák’s approach. Subjective Team-Workload has been compared between the two experimental conditions CoopNT and CoopT performing a Wilcoxon signed-rank test (α = 0.05).

### Subjective cooperation assessment

After completion of each cooperation phase, participants filled in a cooperation questionnaire. It contained six items to quantify perceived cooperation across the different cooperation conditions (Lang *et al.*, 2017). Scores for the six items were averaged and compared between the CoopNT and CoopT conditions performing a Wilcoxon signed-rank test (α = 0.05).

### ECG recording and analysis

The electrocardiogram (ECG) signal was recorded synchronously by means of an electrode fixed on the chest of each participant with a sampling frequency of 256 Hz and referred to the potential recorded at both the earlobes. To emphasize the QRS process, ECG signal was band-pass filtered between 5 and 20 Hz with a 5th-order Butterworth filter. The Pan–Tompkins algorithm (J. Pan and Tompkins, 1985) was employed to find R-waves’ peaks of ECG, and the distance between each two R peaks was measured in seconds. Therefore, RR values were processed to find ectopic interbeat interval.

### EDA recording and analysis

EDA was recorded synchronously through the Shimmer3 GSR+ Unit (Shimmer Sensing, Ireland) by means of two electrodes on the middle and ring fingers of the non-dominant hand for each participant. It was recorded with a sampling frequency of 64 Hz and downsampled to 16 Hz to be processed using the Ledalab suite (Bach, 2014). EDA signal contains a slow-changing part, mostly related to the level of arousal, and a fast-changing part which occurs in relation to single stimuli reactions (Boucsein, 2012). The Continuous Decomposition Analysis (Benedek and Kaernbach, 2010) was applied in order to separate them, respectively, in SCL and skin conductance response. In this analysis, we considered only the SCL signal normalized respect to the baseline value for each subject.

### EEG recording and analysis

The EEG of the dyads has been recorded synchronously by a digital monitoring system (BE+ system, EBNeuro S.p.A., Italy) with a sampling frequency of 256 Hz. For each subject, 25 channels (Fpz, AFz, AF3, AF4, AF7, AF8, Fz, F3, F4, F7, F8, C5, C6, T7, T8, CP3, CP4, CPz, Pz, P3, P4, PO3, PO4, P7 and P8) were referred to both earlobes and their impedances were kept below 10 kΩ. EEG signals were band-pass filtered between 1 and 35 Hz with a 5th-order Butterworth filter, and then a Notch filter has been applied to remove the 50 Hz component influence. Eye-blink contributions were corrected (i.e. without losing data) by REBLINCA algorithm applied to the Fpz channel (Flumeri *et al.*, 2016). The EEG dataset was segmented into epochs of 1 s to respect the EEG stationarity, and for the rejection of artefacts, specific procedures of EEGLAB toolbox (Delorme and Makeig, 2004) were employed. In particular have been marked as artefacts (i.e. samples of the epoch per channel have been assigned NaN) all epochs that did not meet the threshold criterion (±100 μV), the trend estimation (slope was higher than 10 μV/s) or the sample-to-sample criterion (difference, in terms of absolute amplitude, was higher than 25 μV). The percentage of artifacted epoch*channels set to NaN is 23.02 ± 8% for the Alone condition, 23.06 ± 8% for the CoopNT condition and 21.33% ± 9% for the CoopT condition.

For each epoch and each channel, power spectral density (PSD) was calculated using a Hanning window of same length of the considered epoch (1 Hz of frequency resolution). Then, EEG frequency bands (theta, alpha and beta) were defined for each participant accordingly with their individual alpha frequency value (Klimesch, 1999).

### ROIs selection

EEG hyperscanning has been employed to study the natural cooperative behaviour; however, due to the low spatial resolution of this technique, it is not possible to localize the cortical activation associated with cooperation. Cooperation as a social process is mainly associated with the social brain, consisting of brain areas like the medial prefrontal cortex (PFC), orbitofrontal cortex, striatum and amygdala (Gallotti and Frith, 2018). Previous knowledge from fMRI and fNIRS (Functional near-infrared spectroscopy) studies that have higher spatial resolution provides the necessary information regarding both activation and brain mechanisms associated with cooperation. Frontal activity, and in particular, the PFC activity (Wills *et al.*, 2018), is modulated by cooperation because they are components of mentalizing network (Chauvigné *et al.*, 2018). In several fNIRS works, higher coherent PFC activity has been found between actors (Cui *et al.*, 2012; Cheng *et al.*, 2015; Pan *et al.*, 2017). The right temporal cortex (RT) plays a key role in cooperation, proved by several pieces of evidence of high coherence during cooperative tasks (Baker *et al.*, 2016; Ahn *et al.*, 2018). The parietal cortex also is especially expected to be involved due to the theta synchronization in the right parietal cortex and the key role in nonverbal social coordination (Yun *et al.*, 2012). Moreover, the inferior parietal lobule has been proved to be significantly more active during the joint phase respect to alone during joint action paradigms (Egetemeir *et al.*, 2011) and several are the pieces of evidence about high synchronization of temporal and lateral parietal regions in speakers/observer cooperative/tasks (Kawasaki *et al.*, 2013). The hypothesis is that such regions are close to the temporal–parietal junction, the hub of the mentalizing processes (Saxe and Kanwisher, 2003). Therefore, these pieces of evidence have been used to explore the validity of the new approach proposed, and the following nine ROIs have been defined to be tested into the model:

- Frontal area described by the electrodes F3, F4, F7, F8, Fz;
- Right prefrontal cortex (rPFC) defined by the electrodes AF4, AF8, F8;
- Left prefrontal cortex (lPFC) defined by the electrodes AF3, AF7, F7;
- Prefrontal cortex (PFC) defined by the electrodes AF4, AF8, F8, AF3, AF7, F7;
- Left fronto-temporal area (LFT) defined by the electrodes T7, F7;
- Parietal area defined by the electrodes Pz, P3, P4, P7, P8;
- Right centro-parietal area (RCP) defined by the electrodes C6, CP4, P4;
- Right temporo-parietal area (RTP) defined by the electrodes P8, T8;
- Right temporal area (RT) defined by the electrodes C6, T8.

### Time series preparation

For a correct estimation of the multivariate model from each signal and each subject, synchronous neurophysiological time series have been extracted.

The brain dynamics have been obtained averaging PSD values for each band in groups of electrodes belonging to the ROIs identified by the literature review of other hyperscanning studies. Moreover, two complex Neurometrics have been defined to describe each subject in terms of his own mental state:

- Mental Workload as the ratio between PSDs estimated on frontal EEG channels in the theta band and parietal EEG channels in the alpha band (Klimesch, 1999).
- Engagement as the ratio between PSDs estimated on frontal EEG channels in the beta band and the sum of PSDs estimated on frontal EEG channels in the alpha and theta band (Freeman *et al.*, 1999).

If any sample of the time series thus obtained was missed (i.e. was NaN) due to the artefact rejection phase, the missing value was found from a spline interpolation of the nearest epochs.

**Fig. 2.**
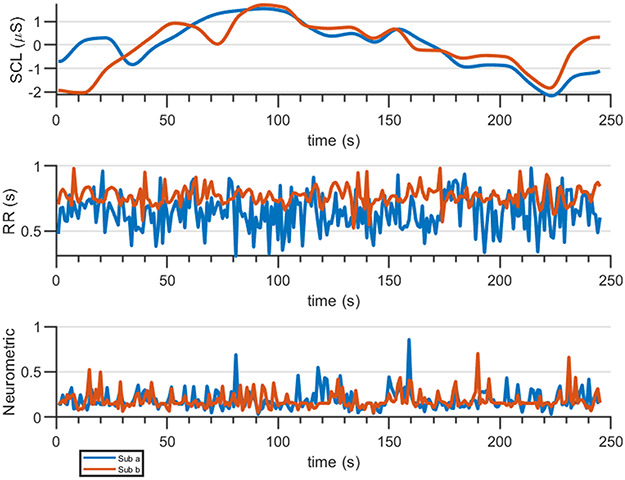
Example of the time series extracted from neurophysiological signals for one couple during the CoopNT condition.

To obtain SCL time series, due to the non-stationarity of the signal, it has been first differentiated twice thus making feasible causality analysis without affecting the results (Barnett and Seth, 2011). Therefore, to synchronize the autonomic signals to brain dynamics they have been both synchronously resampled at 1 Hz using spline interpolation (Zanetti *et al.*, 2019). Finally, each time series has been normalized (Figure 2) and the stationarity has been tested with augmented Dickey–Fuller test (Fuller 2009).

### Multivariate Granger causality analysis

As we have already said, cooperation can be seen as the output of a multivariate system caused by the interaction between behavioural, affective and cognitive mechanisms belonging to two subjects. Therefore, a tool able to measure and explain this interaction could correctly model cooperation. As highlighted so far, the ‘measures’ we are referring to are the neurophysiological time series describing the affective and cognitive state of each subject. To explain interactions between these different components of the system, we employed Granger causality (Granger, 1969) in its conditional form (Geweke, 1984). Let us consider a discrete-time stationary vector stochastic process composed of M real-valued zero-mean scalar processes, ${Y_n} = \left[ y_{1,n} \ldots y_{M,n}\right]^{T}$. Assuming that ${Y_n}$ is a Markov process of order p, in linear signal processing framework it can be completely described by the vector autoregressive model:

(2) \begin{equation*}{Y_n} = \mathop \sum \limits_{k = 1}^p {{\bf{\it{A}}}_k}{Y_{n - k}} + {U_n}\end{equation*}

where ${{\bf{\it{A}}}_k}$ is the M × M matrix containing the autoregressive coefficients ${a_{ij,k}}$ that relate ${y_{j,n}}$ to ${y_{i,n - k}}$$\left( i,j \in \left( {1, \ldots ,M} \right)\right.$, $\left.k \in \left( {1, \ldots ,p} \right) \right)$ and ${U_n} = {\left[{ {{u_{1,n}}} \ldots {{u_{M,n}}}} \right]^T}$is a vector of M zero-mean Gaussian innovation process with covariance matrix ${\boldsymbol\Sigma} = \mathbb{E}\left[ {{U_n}U_n^T} \right]$ (where $\mathbb{E}$ is the expectation value). The problem stated in equation (2) can be solved through the ordinary least square, computing the matrix of coefficients that minimizes the residual error term (Faes *et al.*, 2017).

Let us assume the process ${y_{j,n}}$ as the *target* and ${y_{i,n}}$ as the *driver* process, with the remaining M-2 processes collected in the vector ${Y_{k,n}}$ where $k = \left\{ {1, \ldots ,M} \right\}\backslash \left\{ {i,j} \right\}$. Then, denoting $y_{m,n}^ - = {\left[ {{y_{m,n - 1}},{y_{m,n - 2}} \ldots } \right]^T}$as the past history of the generic process ${y_m}$ we state that the ${i^{th}}$ process G-causes the ${j^{th}}$process (conditional on the other k processes), if $y_{i,n}^ - $ conveys information about ${y_{j,n}}$, above and beyond all information contained in $y_{j,n}^ - $ and $Y_{k,n}^ - $. This definition leads to perform a regression of the present of the target on the past of all processes, yielding to the prediction error ${e_{j|ijk,n}} = {y_{j,n}} - \mathbb{E}\left[ {{y_{j,n}}|Y_n^ - } \right]$, and on the past of all processes except the driver, yielding to the prediction error ${e_{j|jk,n}} = {y_{j,n}} - \mathbb{E}\left[ {{y_{j,n}}|y_{j,n}^ - ,Y_k^ - } \right]$. The prediction error variances resulting from these ‘full’ and ‘restricted’ regressions, ${\lambda _{j|ijk}} = \mathbb{E}\left[ {e_{j|ijk,n}^2} \right]$ and ${\lambda _{j|jk}} = \mathbb{E}\left[ {e_{j|jk,n}^2} \right]$ are then combined to obtain the definition of GC from ${y_i}$ to ${y_j}$:

(3) \begin{equation*}{F_{i \to j}} = ln{{{\lambda _{j|jk}}} \over {{\lambda _{j|ijk}}}}\end{equation*}

In this formulation, the ‘driver’, ‘target’ and conditioning processes may themselves be multivariate and therefore represented by groups of processes. In this sense, it is used the term ‘multivariate’ G-causality for taking into account the group interactions as highlighted in Barnett and Seth (2014). Furthermore, with this formulation, it is possible to take into account the influence of other time series which potentially affect the analysis of the driver and target considered, avoiding the well-known problems related to GC computation in its bivariate formulation (Stokes and Purdon, 2017). All the measures needed for the computation of GC values are performed through MATLAB 2018a and the freely available ITS toolbox (Faes *et al.*, 2015). In this work, two different analyses have been performed.

**Fig. 3.**
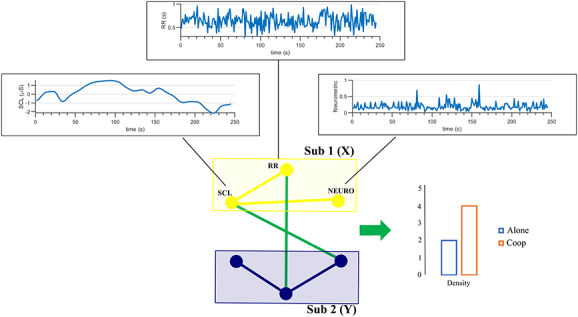
Graphical model of performed analysis for a dyad. Each model has been identified by two groups of time series (SCL: skin conductance level, HR: heart rate, Neuro: Neurometric) each representing a subject. The analysis provided information about the causality within-subject in yellow and in blue, and between-subject in green. The density of connections between subjects has been compared across the different conditions.

In the first analysis, the driver and the target process are alternatively the vectors X and Y representing a group of time series (RR, SCL and a Neurometric) belonging, respectively, to the two subjects. The aim of this step is to select the models significantly affected by cooperation. The GC has been computed for 29 different systems where SCL and RR time series are fixed for all, whereas Neurometric could be Workload, Engagement or each of the time series describing brain activity in ROI for each band (9 × 3). After that, the statistical significance of the estimated GC values has been tested. Surrogate time series have been generated which share the same power spectrum of the original time series but are uncorrelated (Ramseyer and Tschacher, 2011). Specifically, 100 sets of surrogate time series were generated and the corresponding GC were estimated. The 100 values of multivariate GC computed on surrogate time series have been averaged to obtain a distribution of *surrogateGC*. Therefore, for each system and condition there are two different distributions of GC values: *realGC* and *surrogateGC* distribution. These distributions have been statistically compared by means of a paired *t*-test, and the effect size has been computed according to Cohen (1992) to find out if the causality obtained is significant, therefore it is not due to the chance.

The second analysis has been performed only for those systems that showed a significant causal effect due to cooperation. The GC as defined in equation (3) has been computed considering as target process each of the six time series and allowed to obtain the network of the links within and between the neurophysiological time series of each subject (Figure 3). To avoid computational problems associated with the estimation of an empirical distribution with surrogates, in this case, the statistical significance of each estimated causality link has been tested using the asymptotic distribution of GC. In particular, Bernett (2014) showed that under the null hypothesis of zero causality, GC estimator scaled by the sample size has an asymptotic χ^2^ distribution. To quantify the effect due to cooperation for each link, the percentage of subjects showing a significant connection and the density of connections exchanged between subjects have been computed. The density of the links between subjects has been computed as the ratio of the number of existing connections over the total possible amount of connections (Rubinov and Sporns, 2010). The density has been compared between conditions performing a Friedman Test (α = 0.05).

### Correlation analysis

To assess whether there is a relationship between the computed causality and the performance of each dyad, *realGC* of each system which proved to be significantly affected by the effectiveness of cooperation has been correlated with the score obtained in both cooperative phases. A Spearman correlation has been performed.

## Results

### Behavioural results

**Fig. 4.**
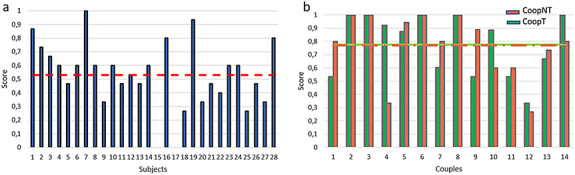
In the left panel, the bars represent the score for each subject. The red line is the mean score of the population. In the right panel, the bars represent the score for each couple during cooperation. The green colour represents the talk condition and the red colour represents the no-talk condition. The lines represent the mean value of score for the talk condition (in green) and the no-talk (in red) respectively.

According to the equation (1), the score has been computed for each condition. During the Alone condition, 2 out of 28 subjects have the lowest performance (subject 15 and subject 17) and only 1 (subject 7) obtains the maximum score. Figure 4a shows the score, and the red line shows the mean score (0.528 ± 0.243).

Figure 4b shows the score for both cooperative conditions. The green bars and the green line represent, respectively, the score during the talk condition (CoopT) for each couple and the average over the population in the same condition (0.777 ± 0.233). The red bars are indicative of the no-talk condition (CoopNT) and the red line of the average over the population in this condition (0.768 ± 0.242). No statistically significant differences have been found (*P *= 0.921).

### Subjective and neurophysiological assessment results

**Fig. 5.**
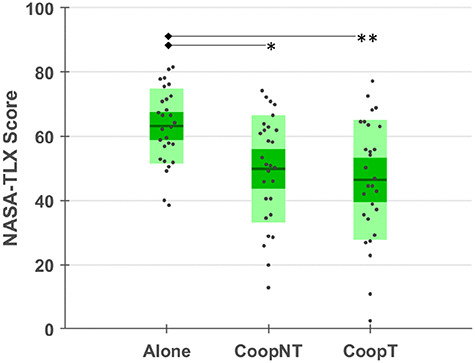
NASA-TLX score for the three conditions. The asterisks show statistical significance for P < 0.05 (*) and for P < 0.01(**).

The NASA-TLX (Figure 5) provides statistically significant differences among the three conditions (χ^2^ = 10.57, *P* = 0.005). The *post hoc* analysis highlights that the Alone condition has significantly higher perceived workload than the cooperative conditions (CoopNT *P* = 0.02; CoopT *P* = 0.009), but there is no statistically significant difference between CoopT and CoopNT (*P *= 0.96).

**Fig. 6.**
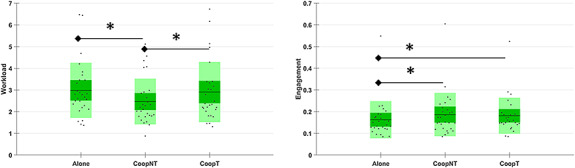
Distributions of Workload and Engagement for each condition. In green, the conditions whose difference is statistically (P < 0.05) significant.

This result has been partially confirmed (Figure 6) by the neurophysiological computation of mental workload (χ^2^ = 21.5, *P* < 0.0001) which shows a significant decrement from Alone condition compared to CoopNT condition (*P *< 0.0001). However, this measure does not identify the difference between Alone and CoopT, but it finds a significant increment of workload during CoopT compared to CoopNT (*P* = 0.006). Moreover, the Engagement shows a significant increment of both cooperative conditions compared to Alone condition (χ^2^ = 7.71, *P *= 0.02; CoopNT *P* = 0.042; CoopT *P* = 0.042).

The two different kinds of interaction during the construction of the bridge have not been perceived significantly different both regarding the difficulty of interaction (*P* = 0.6243) and the quality of cooperation itself (*P* = 0.6751). The results are shown in Table 1.

**Fig. 7.**
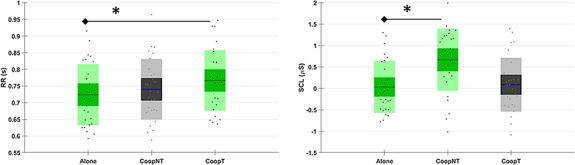
Distributions of autonomic measures (RR, SCL) for the three conditions (Alone, CoopNT and CoopT). In green, the conditions whose difference is statistically (P < 0.05) significant.

From the autonomic point of view (Figure 7), the interbeat interval (RR, χ^2^ = 12.28, *P *= 0.002) shows a significant increment in the case of CoopT respect to the Alone condition (*P* = 0.001), that means lower HR during cooperation. On the other hand, SCL (χ^2^ = 9.5, *P* = 0.008) shows a significant increment during the CoopNT respect to the Alone condition (*P* = 0.009).

The following analyses are organized into two main sections: (i) Multivariate Granger causality analysis and (ii) Analysis of effective and ineffective cooperation.

### Multivariate Granger causality analysis

First of all, time series from neurophysiological signals have been extracted for multivariate GC analysis and ADF test provided *P* < 0.001 for all of them. Table 2 shows the results of the statistical comparison between *realGC* and *surrogateGC* distributions. In no system the Alone condition provides *realGC* values significantly different to *surrogateGC*. In an explorative perspective, 29 possible models have been analysed, and among these, only 7 systems of variables provide significantly higher *realGC* values than *surrogateGC* in both kinds of interactions (CoopNT and CoopT). In each of these cases, the effect size goes from medium (*d* = − 0.595 for PFC Beta in CoopNT) to high (*d* = − 0.907 for Frontal Beta in CoopNT). The results for Workload and Engagement have not been shown in the table because they were not significant under any conditions.

**Table 1.** Results of the questionnaires on team-workload and cooperation

Subjective measures	CoopNT Mean (s.d.)	CoopT Mean (s.d.)	*P-value*	*z*
Team workload assessment	45.77 (11.55)	47.54 (11.75)	0.6243	−0.489
Cooperation assessment	81.96 (16.08)	81.56 (15.00)	0.6751	0.4191

**Table 2.** Results of the empirical validation of real GC values against surrogate GC values per each condition and each system of variables

	Model SCL + RR + ROI		Frontal			PFC			rPFC			lPFC			LFT			RCP			RT			RTP			Parietal		
			θ	α	β	θ	α	β	θ	α	β	θ	α	β	θ	α	β	θ	α	β	θ	α	β	θ	α	β	θ	α	β
	**surrGC**	Mean *std*	0.11 *0.01*	0.11 *0.01*	0.11 *0.02*	0.11 *0.02*	0.11 *0.02*	0.11 *0.02*	0.11 *0.01*	0.11 *0.01*	0.11 *0.01*	0.11 *0.02*	0.11 *0.02*	0.11 *0.02*	0.11 *0.02*	0.11 *0.01*	0.11 *0.01*	0.11 *0.01*	0.11 *0.01*	0.11 *0.01*	0.11 *0.02*	0.11 *0.01*	0.11 *0.01*	0.11 *0.01*	0.11 *0.01*	0.11 *0.01*	0.11 *0.01*	0.11 *0.01*	0.11 *0.02*
Alone																													
	**realGC**	Mean *std*	0.11 *0.03*	0.12 *0.02*	0.10 *0.02*	0.11 *0.03*	0.11 *0.02*	0.11 *0.02*	0.11 *0.02*	0.11 *0.02*	0.11 *0.03*	0.12 *0.03*	0.11 *0.02*	0.11 *0.02*	0.11 *0.02*	0.12 *0.02*	0.11 *0.02*	0.12 *0.02*	0.11 *0.02*	0.11 *0.03*	0.11 *0.03*	0.11 *0.02*	0.11 *0.02*	0.12 *0.03*	0.12 *0.02*	0.11 *0.03*	0.11 *0.03*	0.11 *0.03*	0.11 *0.03*
	***t***		0.38	−0.91	1.30	−0.33	−0.01	−0.08	0.12	0.81	0.09	−1.09	0.04	−0.01	0.08	−0.71	0.04	−0.74	−0.31	0.51	−0.16	−0.07	−0.21	−1.09	−1.07	−0.44	−0.45	−0.56	0.04
	***P***		0.71	0.38	0.22	0.75	0.99	0.94	0.85	0.43	0.92	0.29	0.97	0.99	0.94	0.49	0.96	0.47	0.76	0.62	0.88	0.94	0.84	0.29	0.30	0.67	0.66	0.59	0.97
	**Effect size**		0.10	−0.24	0.35	−0.09	−0.003	−0.02	0.05	0.29	0.03	−0.29	0.01	−0.01	0.02	−0.19	0.01	−0.19	−0.08	0.14	−0.04	−0.02	−0.06	−0.29	−0.29	−0.12	−0.12	−0.15	0.01
	**surrGC**	Mean *std*	**0.13 *0.04***	**0.13 *0.04***	**0.13 *0.05***	0.13 *0.05*	**0.13 *0.05***	**0.14 *0.04***	**0.13 *0.04***	**0.13 *0.04***	**0.13 *0.05***	0.13 *0.05*	**0.13 *0.05***	**0.13 *0.05***	0.13 *0.05*	0.13 *0.05*	0.13 *0.05*	**0.13 *0.05***	**0.13 *0.05***	**0.13 *0.06***	**0.13 *0.05***	**0.13 *0.05***	**0.13 *0.05***	0.13 *0.04*	0.13 *0.05*	**0.13 *0.05***	0.13 *0.05*	0.13 *0.05*	**0.13 *0.05***
**CoopNT**																													
	**realGC**	Mean *std*	**0.15 *0.04***	**0.16 *0.05***	**0.17 *0.06***	0.15 *0.04*	**0.16 *0.05***	**0.17 *0.06***	**0.15 *0.04***	**0.15 *0.05***	**0.16 *0.07***	0.15 *0.04*	**0.16 *0.05***	**0.16 *0.05***	0.14 *0.04*	0.16 *0.06*	0.15 *0.06*	**0.16 *0.03***	**0.16 *0.06***	**0.18 *0.07***	**0.16 *0.04***	**0.17 *0.06***	**0.17 *0.05***	0.15 *0.04*	0.15 *0.05*	**0.16 *0.05***	0.15 *0.04*	0.15 *0.06*	**0.16 *0.07***
	***t***		**−2.59**	**−3.24**	**−3.39**	−1.82	**−2.88**	**−2.23**	**−2.53**	**−2.17**	**−2.29**	−1.49	**−2.88**	**−2.46**	−0.92	−1.64	−1.61	**−2.45**	**−2.38**	**−4.28**	**−2.59**	**−2.89**	**−2.63**	−1.77	−1.56	**−2.49**	−2.09	−1.86	**−2.22**
	***P***		**0.02**	**0.007**	**0.005**	0.09	**0.01**	**0.04**	**0.02**	**0.049**	**0.039**	0.16	**0.01**	**0.03**	0.37	0.13	0.13	**0.03**	**0.03**	**0.001**	**0.02**	**0.01**	**0.02**	0.10	0.143	**0.03**	0.06	0.09	**0.045**
	**Effect size**		**−0.69**	**−0.87**	**−0.91**	−0.49	**−0.77**	**−0.59**	**−0.68**	**−0.58**	**−0.61**	−0.40	**−0.77**	**−0.66**	−0.25	−0.44	−0.43	**−0.65**	**−0.64**	**−1.14**	**−0.69**	**−0.77**	**−0.70**	−0.473	−0.417	**−0.667**	−0.56	−0.49	**−0.59**
	**surrGC**	Mean *std*	0.13 *0.04*	0.13 *0.05*	**0.13 *0.04***	**0.12 *0.04***	**0.13 *0.05***	**0.13 *0.04***	**0.12 *0.04***	0.13 *0.04*	**0.12 *0.04***	**0.12 *0.04***	0.12 *0.04*	**0.13 0.03**	0.12 *0.04*	0.13 *0.04*	0.13 *0.03*	0.13 *0.03*	0.13 *0.05*	0.13 *0.04*	0.12 *0.04*	**0.12 *0.04***	0.13 *0.04*	**0.13 *0.04***	0.12 *0.04*	0.13 *0.04*	0.13 *0.04*	**0.13 *0.05***	0.12 *0.04*
**CoopT**																													
	**realGC**	Mean *std*	0.14 *0.05*	0.15 *0.08*	**0.15 *0.06***	**0.16 *0.07***	**0.16 *0.09***	**0.15 *0.07***	**0.16 *0.06***	0.15 *0.07*	**0.15 *0.07***	**0.15 *0.07***	0.17 *0.15*	**0.15 *0.07***	0.15 *0.07*	0.17 *0.12*	0.14 *0.05*	0.14 *0.06*	0.15 *0.08*	0.14 *0.06*	0.14 *0.06*	**0.15 *0.08***	0.14 *0.08*	**0.15 *0.06***	0.14 *0.07*	0.144 *0.08*	0.14 *0.06*	**0.16 *0.09***	0.13 *0.06*
	***t***		−1.70	−2.14	**−2.57**	**−2.87**	**−2.24**	**−2.36**	**−2.94**	−1.98	**−2.62**	**−2.35**	−1.60	**−2.29**	−2.09	−1.71	−1.56	−1.37	−1.85	−0.82	−1.93	**−2.43**	−1.21	**−2.65**	−2.00	−1.49	−1.50	**−2.22**	−0.94
	***P***		0.11	0.05	**0.02**	**0.01**	**0.04**	**0.03**	**0.01**	0.07	**0.02**	**0.04**	0.13	**0.04**	0.06	0.11	0.14	0.19	0.09	0.43	0.08	**0.03**	0.25	**0.02**	0.07	0.16	0.16	**0.045**	0.37
	**Effect size**		−0.45	−0.57	**−0.69**	**−0.77**	**−0.60**	**−0.63**	**−0.79**	−0.53	**−0.70**	**−0.63**	−0.43	**−0.61**	−0.56	−0.46	−0.42	−0.37	−0.49	−0.22	−0.51	**−0.65**	−0.32	**−0.71**	−0.54	−0.39	−0.40	**−0.59**	−0.25

**Fig. 8.**
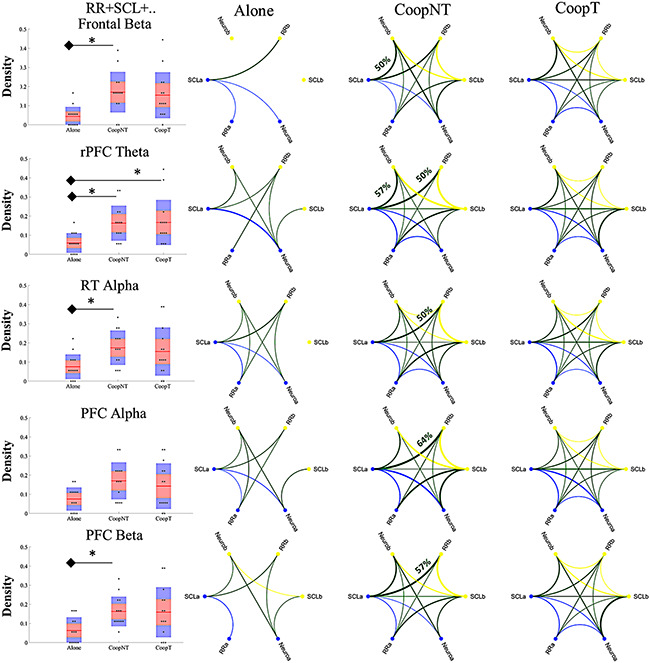
Representation of statistically significant causal relationship between the time series. SCLa, RRa and Neuroa are the time series belonging to the subject a (connections within-subject in blue). SCLb, RRb and Neurob are the time series belonging to the subject b (connections within subject in yellow). In green, the connections between subjects. The thickness represents the percentage of couples having that significant connection. On the left, the distribution for each model of the density of the between-subject connections (the asterisks highlight P < 0.05).

Figure 8 shows the network for each of the five significant models (rPFC and lPFC in beta have been merged into PFC in beta because they were both statistically significant). In particular, for each condition has been shown the circular graph resulting from the computation of pairwise-conditional Granger causality between each time series both within (in blue and yellow for subject a and subject b, respectively) and between time series belonging to different subjects (in green). Only connections significantly different from the chance according to theoretical distribution have been shown and the threshold for visualization has been set on at least two connections. The thickness of the connections shows the percentage of couples having that connection and for each row the maximum value of the percentage reached has been indicated. On the left is shown the density of connections between subjects for each condition. For the system including Frontal beta, the density of the Alone condition is significantly lower respect to the CoopNT (*P *= 0.005, χ^2^ = 10.51). The same is for the density of the model with PFC (*P* = 0.0264) in beta and RT in the alpha band (*P* = 0.016). The rPFC in theta (*P* = 0.003) is also significant compared to the CoopT condition.

### Analysis of effective and ineffective cooperation

**Fig. 9.**
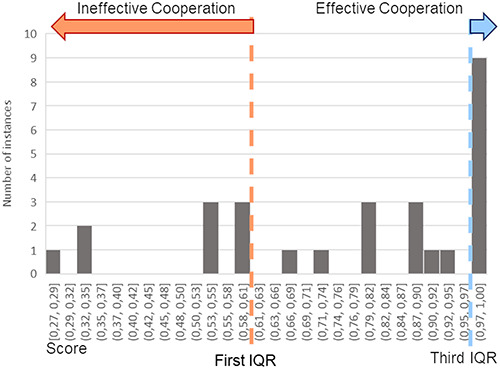
Histogram of scores for cooperative phases. The dashed lines represent the first interquartile range (IQR) in orange and the third IQR in blue, the limits, respectively, of ineffective and effective Cooperation.

Since it has been shown that talk and no-talk condition did not provide any statistically significant difference in both behavioural and perceived measures, all the scores have been used to create a unique distribution. The first and the third interquartile range have been computed to define a threshold for ineffective cooperation and effective cooperation, respectively (Figure 9). The threshold for ineffective cooperation is Score = 0.6 and the threshold for effective cooperation is Score = 1. Eighteen instances have been found in the first quartile and 18 in the third. After labelling each repetition according to this classification, the neurophysiological indexes have been computed again and compared statistically performing a Wilcoxon rank-sum test.

**Fig. 10.**
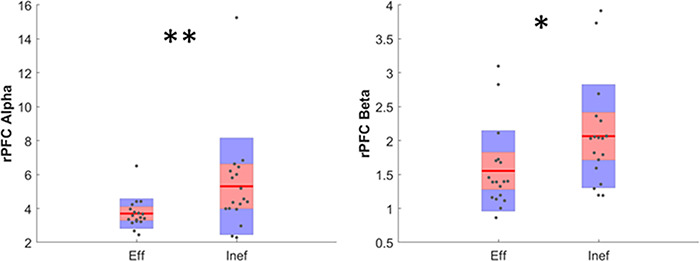
Distribution of PSD values averaged in the rPFC ROI in alpha (left) and beta (right) band. The asterisks define the significance (P < 0.01 ** and P < 0.05 *).

The autonomic signals and Neurometrics have been computed for each condition, and the rank-sum results are shown in Table 3. The autonomic variables, engagement and workload do not differ significantly between these two conditions. The table also shows the results for each ROI in the three different bands analysed. The main significant effects have been found in the beta band: RCP, RT, RTP and parietal activity significantly increase when cooperation is ineffective. The rPFC area is the only one whose activity significantly increases both in alpha and in beta band (Figure 10).

**Fig. 11.**
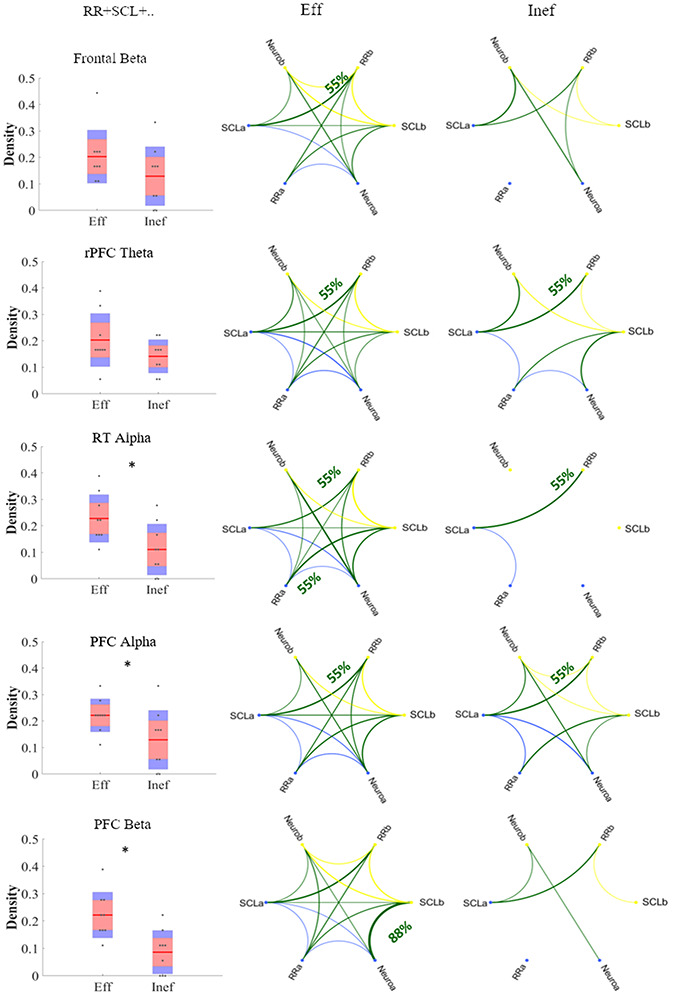
Representation of statistically significant causal relation between the time series in case of effective and ineffective cooperation. SCLa, RRa and Neuroa are the time series belonging to the subject a (connections within-subject in blue). SCLb, RRb and Neurob are the time series belonging to the subject b (connections within-subject in yellow). In green, the connections between subjects. The thickness represents the percentage of couples having that significant connection. On the left, the distribution for each model of the density of the between-subject connections (the asterisks highlight P < 0.05).

Similarly to the previous analysis, we have computed conditional Granger causality considering effective and ineffective classification of the instances. We found (Figure 11) that the density of connections between subjects is significantly lower during ineffective cooperation compared to effective in the PFC model both in alpha (*P *= 0.038) and beta band (*P *= 0.003), in the model with RT in the alpha band (*P* = 0.023) and the rPFC model in the beta band (*P* = 0.007). The other models are not statistically significant.

### Correlation

**Fig. 12.**
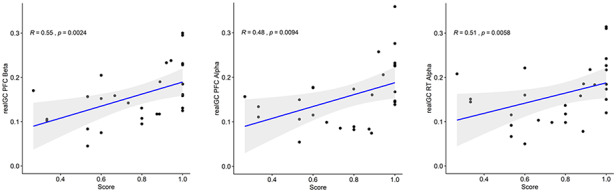
Spearman correlation between the causality values and the score. On the left, the correlation between the model SCL + RR + PFC in alpha and the score. In the middle, correlation between the model SCL + RR + PFC in beta band and the score. On the right, correlation between the model SCL + RR + RT in alpha band and the score.

Figure 12 shows Spearman correlation results between the computed causality for each significant system of variables and the score. In each case, positive medium and significant correlation has been found.

## Discussion

This work aimed at defining a three-dimensional model of cooperation based on neurophysiological measures. In particular, it leveraged the evidence of SPC and hyperscanning and tried to overcome their limitations by analysing interactions between time series extracted from EEG, EDA and ECG signals synchronously recorded from dyads, through state-of-the-art measures of causality. To achieve this goal, we recorded neurophysiological signals during the building of Leonardo’s bridge model, which allowed us to modulate both the level of cooperation (there were both Alone and cooperative phases) and the kind of interaction (no-talk and talk cooperation phases).

Looking at the cooperation phases, the workload level related to the two different levels of interaction and the cooperation itself is not perceived significantly different by subjects (Table 1). However, from both a behavioural (Figure 4) and subjective (Figure 5) point of view, the execution of the task Alone provided lower performances and higher perceived workload than in the cooperative phases. Even if this result could be explained in the framework of joint action according to which sharing a task makes it easier (Wahn *et al.*, 2018, 2020), the influence of learning effects cannot be excluded since no subject performed the task before and the Alone condition was performed firstly to make subjects able to create their own strategy which is a necessary condition for subsequent cooperation. The analysis of neurophysiological workload partially confirms the subjective results with a significantly lower value of workload in no-talk condition but not in talk condition (Figure 6) and the significantly higher value of HR, which is a correlate of the decreased workload (Jorna, 1993), in talk condition compared to the Alone phase (Figure 7). However, the Engagement, i.e. an effortful striving towards task goals which plays a fundamental role in the description of learning phenomena (Matthews *et al.*, 2003), is higher in both cooperative phases compared to the Alone phase (Figure 6). Therefore, we could say that, even if perceived workload decreases, the subjects are still engaged in the task and increasing engagement could be the neurophysiological hint of theorized mental aspects of the joint action, the joint engagement (Seemann, 2009).

**Table 3.** Results of the rank-sum test between effective and ineffective cooperation for neurophysiological variables computed

		RR	SCL	Engagement	Workload				
	*P-value*	0.537	0.476	0.763	0.261				
	*Z*	−0.62	0.711	−0.300	1.123				
ROI		Frontal	rPFC	lPFC	RCP	RT	RTP	LFT	Parietal
${\boldsymbol\theta}$	*P-value*	0.837	0.084	0.739	0.962	0.936	0.886	0.692	0.837
	*Z*	−0.205	−1.724	−0.332	0.047	0.079	−0.142	0.395	−0.205
${\boldsymbol\alpha}$	*P-value*	0.419	**0.009**	0.261	0.457	0.211	0.073	0.401	0.110
	*Z*	−0.806	**−2.578**	−1.123	−0.743	−1.249	−1.787	−0.838	−1.597
${\boldsymbol\beta}$	*P-value*	0.558	**0.016**	0.787	**0.032**	**0.035**	**0.027**	0.401	**0.013**
	*z*	−0.585	**−2.388**	0.268	**−2.135**	**−2.103**	**−2.198**	−0.838	**−2.483**

Finally, no-talk condition induces a significant increment of the tonic component of EDA (Figure 7), that is a reliable indicator of the arousal (Bach *et al.*, 2010) and the stress level of subjects (Posada-Quintero *et al.*, 2016). Therefore, this could be interpreted as a stressor effect because subjects have been forced to cooperate without talking to each other, therefore inhibiting a spontaneous and dominant mechanism (Skoluda *et al.*, 2015).

### Multivariate model

For the sake of interactive mind approach, we defined a multivariate model to derive information from the couple as an interacting entity. This multivariate approach, based on synchronized autonomic and cerebral time series of dyads, allows studying the causality of both affective (from autonomic signals) and cognitive (from brain signals) mechanisms during a cooperative task. Given the novelty of this approach, we decided to analyse, in an explorative way, all the models obtained from the combination of Neurometrics we found of interest in the field of social neuroscience; therefore, in total we analysed 29 models.

The first finding is that none of the multivariate models analysed provided a causal effect higher than the chance level for Alone condition. In contrast, the causal effect is significantly higher than chance level for both cooperation modalities in 7 out of 29 systems of variables explored (Table 2). The relevant Neurometrics are essentially related to activity of the frontal area in beta band as expected for their role in mentalizing processes and team coordination dynamics (Tognoli *et al.*, 2011; Chauvigné *et al.*, 2018). On the one hand, these results fit well in SPC and hyperscanning frameworks, which have already demonstrated higher synchronized autonomic (Elkins *et al.*, 2009b; Ahonen *et al.*, 2018) and cerebral activity (Jahng *et al.*, 2017; Yi *et al.*, 2018) in case of cooperation. On the other hand, this result goes beyond because of the application of multivariate Granger causality to neurophysiological time series that allows to estimate the measure of information exchange in the form of connections between subjects and to validate their existence by applying surrogate testing. Moreover, due to the nature of the task, we were not interested in assessing the direction of this causality because it has been assumed that there was no a predominant role during the task, but the subjects were asked to try to cooperate equally in building the bridge.

Once the causality at dyad-level has been validated identifying the models significantly affected by cooperative behaviour, the analysis of conditional GC allows inferring the topological structure of the network defined by the two subjects. This additional analysis provided both the causal links between the different body districts of the same subject (within-subject network) and those between the different districts of different subjects (between-subject network).

In the within-subject networks, a recurrent pattern between RR, SCL and Neurometric nodes is evident. This result can be explained in the framework of network physiology (Ulke *et al.*, 2017): there is a strong coupling between brain activity in beta band and the autonomic activation of subjects (Kuo *et al.*, 2016). Moreover, this pattern is more frequent during cooperative conditions compared to alone, according to a modulation of brain-peripheral network associated with the task and mental engagement (Zanetti *et al.*, 2019; Antonacci *et al.*, 2020).

In the between-subject networks is evident a statistically significant increment of density, i.e. the number of connections between nodes belonging to different subjects, of the multivariate models involving activity in frontal and PFC in beta, in rPFC in theta and in RT in alpha in the case of cooperation no-talk respect to the Alone phase (Figure 8). Moreover, the most frequent connection between subjects is the one between SCL and RR node of different subjects; therefore, most of the causality is related to autonomic mechanisms.

We can infer that the causal variation of the autonomic time series of two subjects is due to truly interactive and cooperative features of Leonardo’s bridge task, during which subjects automatically perceive their performances which, in a closed-loop, affect their cognitive and affective mechanisms that affect cooperation. Nevertheless, a less immediate interpretation could be made about the links of hybrid nature (e.g. a connection between activity of frontal area in beta band of one subject and HR of the other), but, in general, it is possible to hypothesize that between time series describing the mental and affective state of subjects cooperating, there is a statistical relationship.

### Effective and ineffective cooperation

Once it has been demonstrated that it is possible to find a statistical relationship between time series describing neurophysiological states of subjects cooperating, we analysed whether the proposed approach is valid also to analyse cooperation effectiveness.

The first result is that even during a cooperative task in which subjects are aware of their performance, withdrawal reaction associated with ineffective cooperation is triggered. In fact, the only relevant result in alpha band is the increment of activity in right PFC area during the ineffective cooperation (Figure 10). We can infer that it happens because the subject is aware of his low performance and the right PFC is involved in a system facilitating withdrawal behaviour from aversive sensation (Spielberg *et al.*, 2008). Moreover, there is a significant increment of activity in beta band in right-temporal-parietal areas and, in general, in parietal area in case of ineffective cooperation. This is coherent with previous knowledge that ineffective cooperation induces higher activation of the right hemisphere due to negative valence (Balconi and Vanutelli, 2017a) because it induces feelings more pertinent to competitive behaviour. Adversely, nor autonomic, Workload or Engagement are affected by cooperation effectiveness. Therefore, we can deduce that a variation in engagement could be associated with the cooperative nature of the task and not with the effectivity of the cooperation itself.

The second result, obtained by computing Granger causality, shows that there is a statistical relationship between cooperation effectiveness and exchange of information among nodes of the network. In particular, also in this case, there is a recurrent pattern in the within-subject network, mainly during the effective cooperation, and the density of the between-subject connections was significantly higher during the effective cooperation compared to the ineffective (Figure 11). Moreover, for eight out of nine couples analysed, there is a connection between the frontal brain activity in beta band of one subject and the SCL of the other subject. We hypothesized that this hybrid connection could be a dyad extension of the coupling between brain activity in beta band and the autonomic activation so far demonstrated for the single subject (Kuo *et al.*, 2016).

Therefore, among the models analysed, those involving the PFC in alpha and beta band show to be affected not only by the cooperative nature of the task but also by its effectiveness. In particular, we found that independently of the kind of interactions, the values of Granger causality for significant models provided medium and significant correlation with the pairs’ score (Figure 12). This result is in line not only with SPC framework that found in the synchronization between autonomic signals a correlate of the team performance (Henning *et al.*, 2001) but also with the hyperscanning results that associate brain network properties with cooperation effectiveness (Sciaraffa, N., Borghini, G., Aricò, P., *et al.*, 2017). At the same time, however, this result goes beyond because it has been obtained integrating information from both autonomic and cognitive mechanisms.

## Limitations and conclusions

To the best of our knowledge, this work is the first example of synchronized autonomic and brain signals of dyads performing a cooperative task analysed in a unique multivariate model. The pros of this approach could be summarized in multifaceted analysis of cooperation (affective mechanisms analysed by means of autonomic signals, cognitive mechanisms by means of EEG) without failing in the analysis of non-stationary time series and coincidental synchrony, but with an attempt of validating the causality emerged from the task.

However, also this research showed some limitations. First, the number of couples investigated is limited and needs to be improved, even if the interactive minds approach for its own nature is penalized respect to the isolated minds. Second, due to the novelty of this work, we have chosen to perform an exploratory study of all the models obtained from the combination of Neurometrics we found of interest in the field of social neuroscience, and therefore, we have not corrected statistical analysis for multiple comparisons according to Rothman (1990). Moreover, even if the surrogate time series have been created to assess the estimated links in the network, this is just one of the possible approaches that can be used. However, the empirical approaches like this have to be preferred to theoretical since they are less influenced by effect size (Hacker and Abdulnasser, 2006). For a more complete model also other biosignals should be taken into account. However, this is not easily implementable due to methodological limitations: the accuracy of causality analysis is strongly influenced by the ratio between the number of time series and the number of data samples available (Antonacci *et al.*, 2017, 2019). Finally, the ROIs have been selected from a literature review of different hyperscanning works and then translated in the most adequate EEG channels. This procedure is biased by the low number of EEG channels employed. An optimal approach should use a combined EEG and fNIRS system or high-resolution EEG in order to overcome the low spatial resolution related issues.

Taking these limitations into account, the results remain noteworthy because they have shown that both cooperation and the kind of interaction affect the neurophysiological condition of subjects involved in the task. For the first time, neurophysiological effects of joint engagement at the basis of joint action have been observed during the cooperative task, alongside stressful effects of obstructive cooperation. Finally, the multivariate approach shows that only during cooperation there is a causal relationship significantly different by the chance level between the time series describing the cognitive and emotional state of each dyad and that the entity of this causality is correlated to the effectiveness of cooperation itself.
